# P2X7 Receptor Antagonism as a Potential Therapy in Amyotrophic Lateral Sclerosis

**DOI:** 10.3389/fnmol.2020.00093

**Published:** 2020-06-12

**Authors:** Cristina Ruiz-Ruiz, Francesco Calzaferri, Antonio G. García

**Affiliations:** ^1^Instituto Teófilo Hernando and Departamento de Farmacología, Facultad de Medicina, Universidad Autónoma de Madrid, Madrid, Spain; ^2^Instituto de Investigación Sanitaria, Hospital Universitario de La Princesa, Universidad Autónoma de Madrid, Madrid, Spain

**Keywords:** amyotrophic lateral sclerosis, ALS, neuroinflammation, P2X7, P2X7 receptor antagonists, calcium dyshomeostasis, mitochondrial disruption, motoneuron death

## Abstract

This review focuses on the purinergic ionotropic receptor P2X7 (P2X7R) as a potential target for developing drugs that delay the onset and/or disease progression in patients with amyotrophic lateral sclerosis (ALS). Description of clinical and genetic ALS features is followed by an analysis of advantages and drawbacks of transgenic mouse models of disease based on mutations in a bunch of proteins, particularly Cu/Zn superoxide dismutase (SOD1), TAR-DNA binding protein-43 (TDP-43), Fused in Sarcoma/Translocated in Sarcoma (FUS), and Chromosome 9 open reading frame 72 (C9orf72). Though of limited value, these models are however critical to study the proof of concept of new compounds, before reaching clinical trials. The authors also provide a description of ALS pathogenesis including protein aggregation, calcium-dependent excitotoxicity, dysfunction of calcium-binding proteins, ultrastructural mitochondrial alterations, disruption of mitochondrial calcium handling, and overproduction of reactive oxygen species (ROS). Understanding disease pathogenic pathways may ease the identification of new drug targets. Subsequently, neuroinflammation linked with P2X7Rs in ALS pathogenesis is described in order to understand the rationale of placing the use of P2X7R antagonists as a new therapeutic pharmacological approach to ALS. This is the basis for the hypothesis that a P2X7R blocker could mitigate the neuroinflammatory state, indirectly leading to neuroprotection and higher motoneuron survival in ALS patients.

## Introduction

Amyotrophic lateral sclerosis (ALS) is a progressive neurodegenerative disease with upper and lower motor neuron (MN) loss in the cerebral cortex, brainstem, and spinal cord. Clinical symptoms start at 55 ± 15 years, with gradual loss of daily motor activities, ambulation, speech and swallowing impairment, compromised respiration, progressive paralysis, and death within 3–5 years after diagnosis. Due to this prompt death, disease prevalence is low; in the UK, the incidence is 1 in 472 women and 1 in 350 men (Alonso et al., 2009), while in the USA, there are about 16,000 affected patients and 5,000 new diagnosed cases every year (The ALS association, 2020).

For the last 25 years, the only pharmacological treatment available has been riluzole, a drug that blocks glutamate release from presynaptic terminals and accelerates glutamate clearance from the synapse. Riluzole also elicits partial blockade of presynaptic sodium channels and inhibits postsynaptic N-methyl-D-aspartate (NMDA) receptors (Doble, 1996). Unfortunately, riluzole is only able to prolong patients’ life for approximately 2 months (Miller et al., 2003).

A second drug, edaravone, was approved by the U.S. Food and Drug Administration (FDA) in 2017, with antioxidant effects that presumably preserve MN viability (Cruz, 2018). This approval generated great controversy inasmuch as it was based in a single clinical trial (CT) performed in Japanese patients, with genetic background distinct from that of Caucasian patients. Furthermore, ALS incidence is lower in Asian population, compared with European/American population (Chio et al., 2013). In any case, it is worth mentioning that edaravone has already been approved in Japan (2015), South Korea (2015), USA (2017), Canada (2018), Switzerland (2019), and China (2019).

In light of the scarce therapeutic tools currently available to delay the appearance of symptoms or disease progression, we will analyze here the following aspects of ALS to be able to extract meaningful conclusions for new drug therapy approaches: (i) genetic features; (ii) mouse models of ALS, based on gene mutations found in patients; (iii) pathogenic signaling pathways involved in MN death, and potential drug targets; (iv) neuroinflammation and the role of the purinergic P2X7 receptor (P2X7R) in disease pathogenesis; (v) available ligands for P2X7Rs; (vi) proof of concept of efficacy of some P2X7R antagonists in mouse models of ALS; (vii) past and ongoing CTs; and (viii) conclusions and perspectives.

## Genetics of ALS

A family history is present in 5–10% of patients of ALS (familial ALS or fALS). The first mutations reported were those in the gene coding for Cu/Zn superoxide dismutase (SOD1) protein, which account for around 15% of all fALS and 1–2% of the sporadic cases (sporadic ALS or sALS) in European population (Rosen et al., 1993; Stephenson and Amor, 2017; Zou et al., 2017). Interestingly, these mutations (nearly 200 different mutations have been reported) do not impair the enzymatic activity of SOD1. Rather, they cause its pathological aggregation in the cytosol or within mitochondria, leading to MN toxicity (Monk and Shaw, 2006). The observation that all cell types express SOD1, yet only MNs degenerate, is puzzling (Pasinelli and Brown, 2006). This could be due to the low capacity of Ca^2+^ buffering exhibited by these neurons (Bezprozvanny, 2009; De Diego et al., 2012).

Around 40 different mutations in the *TARDBP* gene, which encodes for the transactive response (TAR)-DNA binding protein-43 (TDP-43), have been described. Mutations account for the 4–5% of fALS and <1% of sALS in European population (Arai et al., 2006; Neumann et al., 2006; Zou et al., 2017). These mutations cause the aggregation of TDP-43 protein in the cell cytoplasm, which has important functions in RNA processing and regulation. Interestingly, 97% of ALS patients present TDP-43 inclusions that are only absent in patients carrying SOD1 mutations, revealing the high relevance of this protein in ALS pathogenesis.

Mutations in the Fused in Sarcoma/Translocated in Sarcoma (*FUS/TLS*) gene have also been linked to the pathogenesis of ALS (Lagier-Tourenne and Cleveland, 2009). Mutations in FUS are present in 3% of fALS and <1% of sALS European patients (Zou et al., 2017), and they are responsible for the early-onset forms of the disease. This protein has relevant functions in RNA splicing and transport, and it also forms aggregates in the cell cytoplasm of ALS patients with this mutation.

Hexanucleotide repeats (GGGGCC) in intron 1 of chromosome 9 open reading frame 72 (*C9orf72*) gene were identified as the major cause of ALS, being present in 30–40% of fALS and 5% of sALS cases in European population (DeJesus-Hernandez et al., 2011; Renton et al., 2011; Majounie et al., 2012; Zou et al., 2017). Hexanucleotide repeats seem to cause RNA toxicity due to the expansion of sense and antisense RNAs, the aggregation of RNA binding proteins, and the translation of repeat-association non-ATG (RAN) proteins. Patients harboring these mutations also present TDP-43 protein inclusions in the cytoplasm (Liu Y. et al., 2016).

## Modeling ALS Genetics in Mice

The ALS mutations mentioned above have been identified as leading causes of disease, and based on them, several mouse models have been developed. Ideally, mouse models should present all the hallmarks of the human disease, namely, MN loss, muscle weakness and atrophy, metabolic deficits, TDP-43 inclusions and proteinopathy, gliosis, and changes in innate immunity. Even though available models are far from being perfect, they have been useful to understand ALS pathogenesis, to identify new drug targets with therapeutic potential, as well as to establish the proof of concept of the efficacy and safety of lead compounds (Stephenson and Amor, 2017). This is particularly true for the SOD1^G93A^ mice that is by far the model that has been, and it is being used more frequently.

The discovery of new genes linked to ALS (Chia et al., 2018) and the fast development of gene editing techniques such as CRISPR/Cas9 will surely increase the number of ALS mouse models, giving rise to a drug testing platform that includes the high variability of the human disease. We describe below the models that are more frequently used, emphasizing their advantages and caveats for drug discovery, previously described in two detailed recent reviews (Stephenson and Amor, 2017; Lutz, 2018).

### SOD1 Models

SOD1 overexpressing mouse models were developed in the first place, and they are still the most used when it comes to ALS drug discovery (Rosen et al., 1993; Gurney et al., 1994). In particular, the SOD1^G93A^ mouse model has been the most used and studied of all of them, as it closely resembles the disease progression in humans. These mice develop a progressive MN disease with adult onset and reduced lifespan. Pathogenically, SOD1^G93A^ mice show early onset astrogliosis and microgliosis, glutamate-induced excitotoxicity, deficits in axonal transport, axonal denervation, protein aggregation, aberrant neurofilament processing, and mitochondrial vacuolization. All these processes result in the selective loss of spinal cord MN and the marked wasting, paralysis and atrophy of the forelimbs and hindlimbs. Thus, this model exhibits almost all the hallmarks of ALS (Philips and Rothstein, 2015; Lutz, 2018).

However, some disadvantages are found in this model. As mutations in SOD1 are found in a minority of ALS cases, the model is not representative of the whole patient population. This is of great importance, as SOD1 mutations do not result in TDP-43 inclusions in the SOD1^G93A^ model, with this hallmark of disease pathogenesis being not commonly present in either SOD1 patients or mice (Stephenson and Amor, 2017). However, other SOD1 models such as SOD1^G86S^ do exhibit TDP-43 inclusions and could be used to overcome this pitfall (Jeon et al., 2019). Furthermore, while patients develop the disease with basal quantities of the mutated protein, mice show very high overexpression of the mutated SOD1 protein (Lutz, 2018). In addition, in mice, the disease always commences in lumbar spinal cord MNs, while in humans, ALS onset may be initiated in cortical, bulbar, or spinal cord MNs (Philips and Rothstein, 2015).

### TDP-43 Models

Mouse ALS models based in *TARDBP* mutations, encoding for TDP-43 protein, have failed in most cases to mimic the human disease, the overexpressing model TDP-43^A315T^ being the best one in resembling it. These mice show an adult onset, with progressive and fatal neurodegeneration. Mice present TDP-43 aggregates, activation of astroglia and microglia, as well as MN loss. However, survival is impaired by a severe gastrointestinal pathology that can cause their sudden death previous to neurodegeneration (Wegorzewska et al., 2009; Herdewyn et al., 2014). Further limitations of the model are the overexpression of mutated TDP-43 and a poor loss of spinal cord MNs.

### FUS Models

The majority of mouse models based in human *FUS* mutations do not present a complete ALS-like phenotype, neither progression to paralysis or disease end stage. The model that more closely resembles fALS is the wild type (WT) overexpressing mouse or hFUS^WT^. Mice develop paralysis, weight loss, muscle atrophy, and tremor. Moreover, MN loss, protein aggregation, and gliosis are also present. The major caveat of the model is that it does not carry a disease-linked mutation and, hence, the underlying pathology may not be the same as in the human disease (Mitchell et al., 2013).

### C9orf72 Models

Again, most C9orf72-based models failed to develop an ALS-like pathology. The most suitable model is the h(G_4_C_2_)_37-500_, which shows muscle weakness, paralysis, weight loss, cognitive deficits, and premature death. Moreover, mice show histopathological hallmarks of the disease, such as MN loss, TDP-43 proteinopathy, and gliosis. However, this pathology is only developed by a 30–35% of female subset of mice. Another 40% of females and around 45% of males develop a milder pathology that in some cases progresses to the disease end point (Liu Y. et al., 2016). This heterogeneity seriously limits the use of this model to test the effect of compounds within a drug development program.

## ALS Pathogenesis

ALS pathogenesis is far from being completely understood. Even though the disease is known to be multifactorial and multisystem, it is still unknown why the selective death of MNs occurs. Our limited knowledge on disease pathogenesis could explain why over 50 CTs with a large variety of compounds targeting different receptors and signaling pathways have provided negative outcomes in ALS patients. We will review here some features of disease pathogenesis such as cell Ca^2+^ dyshomeostasis, mitochondrial Ca^2+^ handling, and production of reactive oxygen species (ROS). Neuroinflammation and the role of P2X7 receptor (P2X7R) will be commented in “Neuroinflammation and P2X7 Receptors in ALS Pathogenesis” section.

### Calcium Dyshomeostasis: The Excitotoxic Hypothesis

The excitotoxic hypothesis implies that alterations of some of the multiple receptors, ion channels, and calcium-binding proteins (CBPs), could be the result of some of the ALS mutations above described and the aggregation of pathological proteins (Appel et al., 2001; Van Den Bosch et al., 2006; Bezprozvanny, 2009; Grosskreutz et al., 2010; De Diego et al., 2012). Several factors support the assumption that augmented glutamatergic neurotransmission and Ca^2+^-dependent excitotoxicity play central relevant roles in MN degeneration (Patai et al., 2017). They are as follows: (i) high levels of excitatory amino acids have been found in the cerebrospinal fluid (CSF) of ALS patients (Plaitakis and Caroscio, 1987; Rothstein et al., 1991; Fiszman et al., 2010); (ii) CSF from ALS patients exerts MN death *in vitro* (Terro et al., 1996; Sen et al., 2005; Anneser et al., 2006; Gunasekaran et al., 2009; Yáñez et al., 2011); (iii) selective loss of astroglial excitatory amino acid transporter 2 (EAAT2) in motor cortex and spinal cord is found in both sporadic and fALS (Rothstein et al., 1995; Fray et al., 1998; Lin et al., 1998; Sasaki et al., 2000) with concomitant impairment of astrocytes’ ability to remove glutamate from the synaptic cleft; (iv) a defective glutamate clearance was detected in synaptosomes from brain areas and spinal cord of sALS patients (Rothstein et al., 1992), but prolonged *in vivo* blockade of astroglial EAAT2 with concomitant extracellular glutamate increase was innocuous for spinal MNs in rats (Tovar et al., 2009); and (v) α-amino-3-hydroxy-5-methyl-4-isoxazolepropionic acid receptors (AMPARs) rather than NMDARs seem to contribute more to MN cytotoxicity, firstly because they are expressed at higher density in cultured MNs (Carriedo et al., 1996; Van Den Bosch and Robberecht, 2000) and, secondly, because of their higher Ca^2+^ permeability due to the lack of a GluR2 subunit in the tetrameric receptor complex (Van Damme et al., 2002). Consistent with this are the observations of lower GluR2 mRNA levels in MNs compared with other neurons (Van Damme et al., 2002), and the lower AMPAR mRNA in human spinal MNs (Heath and Shaw, 2002). Furthermore, the lack of GluR2 in SOD1^G93A^ mice accelerated MN loss (Van Damme et al., 2005), and conversely, GluR2 overexpression augmented MN lifespan (Tateno et al., 2004). Of physiological and pharmacological interest are the observations that the GluR2 subunit is regulated by the vascular endothelial growth factor (VEGF; Bogaert et al., 2010) as well as by the brain-derived neurotrophic factor (BDNF) and glia cell-derived neurotrophic factor (GDNF; Brené et al., 2000). Of note is the fact that these growth factors also contribute to maintain MNs in good health. This is the case for VEGF (Wang et al., 2016), BDNF (Lanuza et al., 2019), and GDNF (Thomsen et al., 2018).

Strong or weak capacity to bind free Ca^2+^ ions in the cytosol ([Ca^2+^]_c_) by CBPs has immediate consequences for neuron vulnerability during Ca^2+^ loads in ALS-linked MN degeneration. For instance, cortical, lower cranial, and spinal MNs die soon after ALS onset, which correlates with their lack of expression of the CBPs parvalbumin and calbindin-D28k. In contrast, MNs from Onuf’s nucleus, oculomotor, trochlear, and abducens nerves that express high levels of parvalbumin and/or calbindin-D28k are resistant to damage or are injured at advanced disease stages (Alexianu et al., 1994). Moreover, in brain slices, the expression level of CBPs by different types of neurons correlate with their specific vulnerability to Ca^2+^ loads (Ho et al., 1996; Beers et al., 2001; Van Den Bosch et al., 2002). In line with these results, another experiment showed that cross breeding SOD1 mutant mice with mice overexpressing parvalbumin in spinal MNs resulted in a delay of disease onset and a longer MN survival (Beers et al., 2001).

### Altered Mitochondrial Calcium Handling

Mitochondria are involved in nearly all types of cell death, including necrosis, apoptosis, and necroptosis (Kroemer et al., 1998; Galluzzi and Kroemer, 2008). But notably, the redistribution of mutated TDP-43 from the nucleus to the cytoplasm is being considered a pathological hallmark for most forms of ALS (Kabashi et al., 2008; Sreedharan et al., 2008). A recent study demonstrates that TDP-43 toxicity resides in its mitochondrial location. The aberrant protein specifically impairs the Complex I of the mitochondrial oxidative phosphorylation system (OXPHOS) by preferentially binding to mitochondrial transcribed ND3/6 mRNAs as well as by inhibiting their translation, leading to mitochondrial dysfunction and neurodegeneration. The interest of this study resides in the additional observation that the suppression of WT or mutant TDP-43 in mitochondria restores neuronal viability. This provides a rationale for reducing the aggregated TDP-43 protein inside the mitochondria as a therapeutic approach for ALS (Wang et al., 2016).

Moreover, mitochondria in MNs from SOD1^G93A^ and TDP-43 mice show a swelling shape, have internal vacuoles and disorganized cristae, and aggregate in abnormal clusters. This causes a defect in mitochondrial axonal transport towards the synaptic nerve terminals, leading to neuronal metabolic alterations (Magrane et al., 2014). Mitochondria also show aberrant fission and fusion dynamics that are necessary for damaged mitochondria confinement and repair (Smith et al., 2019).

### The Link Between ROS and Mitochondrial Calcium Cycling

ROS are natural metabolic products of cell functions and particularly of oxidative phosphorylation itself. However, impairment of mitochondrial electron transport chain generates an excess of ROS that can damage cell proteins, membrane lipids, and nucleic acids. In fact, several markers of ROS damage, i.e., 3-nitrotyrosine, 8-hydroxy-2′-deoxyguanosine, and 4-hydroxynonenal, have been found in blood, CSF, and urine of ALS patients, as well as in their spinal MNs (Smith et al., 1998; Ihara et al., 2005; Mitsumoto et al., 2008).

Oxidative stress is also caused by a defect in the cellular defense machinery against ROS. Such is the case of SOD1 that catalyzes the conversion of superoxide radicals (O_2_^−^) into hydrogen peroxide (H_2_O_2_). Physiological reduction of H_2_O_2_ into oxygen and water through the action of glutathione and catalase offers cell protection from ROS. Interestingly, the mutation of SOD1 in ALS does not result in a loss of its enzymatic function, but rather, it enhances its ability to oxidize cellular antioxidants, reducing molecular oxygen to O_2_^−^ (Liochev and Fridovich, 2003). Nevertheless, the MN toxicity linked to mutated SOD1 is mainly due to the cytoplasmic aggregates created by the aberrant protein (Hardiman et al., 2017).

Large Ca^2+^ loads occurring upon cell stimulation are cleared up by mitochondria through the high-capacity mitochondrial Ca^2+^ uniporter (MCU; Herrington et al., 1996; Xu et al., 1997; Montero et al., 2000). The Ca^2+^ accumulated in the mitochondrial matrix stimulates respiration and ATP synthesis, to couple cell activity and bioenergetics needs (Gunter et al., 1994; Rizzuto et al., 2000). During the excitotoxic process, this accumulation could lead to excessive mitochondrial Ca^2+^ load, oxidative stress, and cell death (Cano-Abad et al., 2001; Orrenius et al., 2003).

Correlation between Ca^2+^ influx with massive mitochondrial Ca^2+^ uptake also occurs in hypoglossal MNs (Ladewig et al., 2003). Such mitochondrial Ca^2+^ uptake is comparatively small in highly Ca^2+^-buffered ALS-resistant spinal MNs (Carriedo et al., 2000), suggesting that mitochondria could partially compensate for the weak cytosolic Ca^2+^ buffering in vulnerable neurons. In fact, following Ca^2+^ entry through voltage-activated calcium channels (VACCs), substantial mitochondrial Ca^2+^ uptake occurs in weakly buffered neurons (Bergmann and Keller, 2004). Mitochondria may exert an additional control of Ca^2+^ homeostasis through ROS-dependent regulation of excitability. Thus, enhanced ROS production occurs upon inhibition of respiration by sodium cyanide. ROS induces the opening of sodium channels, augmentation of action potential firing and enhanced voltage-dependent Ca^2+^ influx (Bergmann and Keller, 2004). Some studies support the idea that the production of ROS is increased in spinal MN mitochondria as a result of Ca^2+^ overload following excitotoxic stimulation of AMPA/kainate receptors (Carriedo et al., 2000).

Mitochondrial damage elicited by mutant SOD1 aggregates found in the mitochondrial matrix of transgenic mice (Jaarsma et al., 2001; Pasinelli et al., 2004) could decrease the enzymatic activity of the electron transport chain at complexes I, II, and IV (Jung et al., 2002; Mattiazzi et al., 2002). In fact, mutant SOD1 could disrupt the association of complex IV (cytochrome c) with the inner mitochondrial membrane, thereby interfering with respiration (Kirkinezos et al., 2005). This leads to increased ROS production as shown in mutant SOD1-expressing MNs cultures (Kruman et al., 1999) as well as in MNs of brain slices where complex IV was inhibited by sodium cyanide (Bergmann and Keller, 2004).

It seems that ROS produced in MNs can diffuse to neighboring astrocytes to cause oxidative disruption of glutamate transporters (Rao et al., 2003). In turn, this will increase the local extracellular concentration of glutamate, thereby enhancing local excitotoxicity in a vicious circle of MN damage. This model integrates the hypothesis of Ca^2+^-dependent excitotoxicity and oxidative damage. In this frame, distorted mitochondrial respiration sensitizes MNs to glutamate stimulation and to environmental toxins, thus increasing their vulnerability (Kruman et al., 1999; Andreassen et al., 2001). In this direction, it was observed that in cultured neurons, the chronic mitochondrial inhibition induced by malonate or sodium azide led to selective MN death; free-radical scavengers and AMPAR blockers protected such neurons from death (Kaal et al., 2000).

## Neuroinflammation and P2X7 Receptors in ALS Pathogenesis

The purinergic P2X7 ionotropic receptor (P2X7R) is considered as one of the main players of inflammation (Di Virgilio, 2015). In fact, this receptor is expressed in immune and inflammatory cells such as dendritic cells, osteoclasts, microglia, mast cells, natural killer cells, or T and B lymphocytes. Additionally, P2X7Rs are upregulated in inflammatory processes (Ferrari et al., 2006).

As a result of cell stress or tissue damage, large amounts of ATP are released into the extracellular space that stimulates the low-affinity target P2X7R, the main sensor for ATP during inflammation. This receptor is also the main trigger of the protective/regenerative immune response, consisting in the maturation and release of several interleukins, mainly IL-1β (Adinolfi et al., 2018).

The maturation of IL-1β requires the activation of the NLRP3 inflammasome, an event that requires Na^+^ influx accompanied by water, Ca^2+^ influx, and K^+^ efflux. The intracellular K^+^ drop is, in fact, the best established mechanism underlying the P2X7R-mediated formation of the NLRP3 inflammasome (Compan et al., 2012; Katsnelson et al., 2015; Jo et al., 2016; Karmakar et al., 2016). This is supported by experiments showing that not only ATP, but also other agents that induce K^+^ efflux, such as the ionophore nigericin or crystalline molecules, elicit inflammasome activation too (Compan et al., 2012; Yaron et al., 2015). Noteworthy, ROS, also, induce inflammasome aggregation and IL-1β release due to P2X7R activation (Hung et al., 2013; Minkiewicz et al., 2013).

The role of P2X7Rs in neuroinflammation is supported by the fact that they are expressed in various cells of the central nervous system (CNS), namely, astrocytes, microglia, and oligodendrocytes (Sperlagh et al., 2006; Kaczmarek-Hajek et al., 2018), where they mediate inflammasome signaling (Franceschini et al., 2015). The presence of reactive astrocytes and microglia define the neuroinflammatory process in the CNS. As peripheral immune and endothelial cells, activated microglia and astrocytes produce pro-inflammatory cytokines (IL-1β, IL-16, TNF-α), chemokines (CCL2, CCL9, CCL1), ROS, and secondary messengers (nitric oxide, prostaglandins; DiSabato et al., 2016). However, the activation of P2X7Rs in these cells is known to produce dual and divergent effects depending on the duration of the inflammatory stimulus and the disease stage, presymptomatic or symptomatic.

How neuroinflammation contributes to neurodegeneration has recently been analyzed by Ransohoff (2016). Thus, a chronic mild activation of glial cells *via* P2X7Rs leads, in the long term, to synaptic dysfunction, synapse loss, and neuronal death. The fact that glial cells exhibit a low overall turnover rate make them more susceptible to the neuroinflammatory effects of age, injury, or stress in neurodegenerative diseases (Ajami et al., 2007).

A dual effect of P2X7R-mediated neuroinflammation seems to operate during the time course of ALS. This could be explained in the context of two different phenotypes that microglia develop upon P2X7R activation. The M2 phenotype or anti-inflammatory predominates at earlier disease stages, while the pro-inflammatory M1 phenotype is prevalent at later disease stages in SOD1^G93A^ mice (Liao et al., 2012; Parisi et al., 2016). Consistent with this is an experiment done in SOD1^G93A^ mice with genetic ablation of P2X7Rs; exacerbation of gliosis and enhanced MN death were unexpectedly found in these mice (Apolloni et al., 2013a). Another experiment supporting duality indicated that short P2X7R stimulation augmented autophagy and M2 microglial markers. However, continued P2X7R stimulation impaired autophagy, suggesting a microglial shift to the M1 phenotype. A dual report that implies duality of action at central and peripheral tissues indicated a positive role of P2X7Rs on SOD1^G93A^ muscles (Fabbrizio et al., 2020).

A few preclinical and clinical experiments support the implication of P2X7Rs in ALS pathogenesis. So, the pro-inflammatory action of microglial P2X7Rs was augmented in SOD1^G93A^ mice (D’Ambrosi et al., 2009). Furthermore, spinal cord pathology was ameliorated by P2X7R antagonism, also in these mice (Apolloni et al., 2014). Consistent with this was the observation that P2X7R activation by high ATP concentrations activated NOX2 and the kinase ERK1/2 in the microglia of SOD1^G93A^ mice, thus provoking an increase of ROS (Apolloni et al., 2013b). Additionally, in co-cultures of astrocytes and MNs from SOD1^G93A^ mice, cell stimulation with P2X7R agonists ATP and 3′-O-(4-Benzoyl)benzoyl ATP (BzATP) elicited a neurotoxic phenotype that was prevented by the P2X7R blocker Brilliant Blue G (BBG; Gandelman et al., 2010). However, unexpected P2X7R down-regulation and Ca^2+^ dyshomeostasis was found in peripheral monocytes of ALS patients (Liu J. et al., 2016). This contrasts with another study showing up-regulation in spinal cord tissue of post-mortem ALS patients (Yiangou et al., 2006).

This cumulative set of data mostly from preclinical *in vitro* and *in vivo* models of ALS, plus a scarce number of clinical studies in ALS patients, support the view in the sense that P2X7Rs mediate the activation of astrocytes and microglia, giving rise to a chronic neuroinflammatory state that is critical in the progression of ALS pathology. However, it should be kept in mind that dependent on disease stage, microglial activation may be anti-inflammatory (early stage, M2 phenotype) or pro-inflammatory (late stage, M1 phenotype).

## Some P2X7R Blockers With Favorable Pharmacokinetics

**Figure 1:**
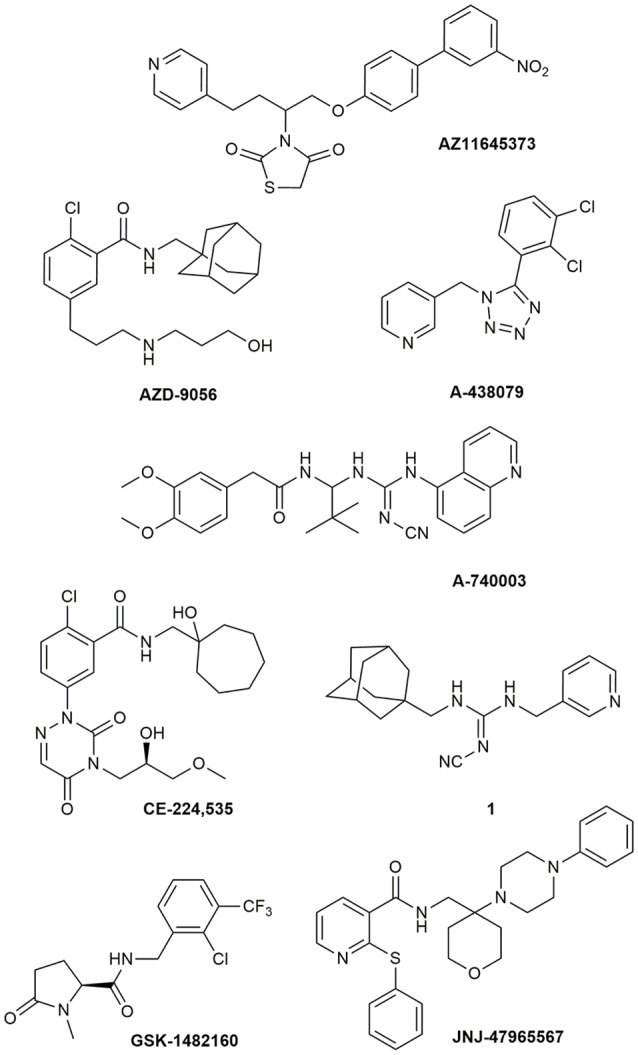
P2X7R antagonists lacking adequate pharmacokinetic properties for the treatment of CNS disorders.

Due to its involvement in neuroinflammation, the P2X7R is considered an adequate target for the treatment of several neurodegenerative and neurological diseases, as well as mood disorders (Díaz-Hernández et al., 2009; Hempel et al., 2013; Bhattacharya and Biber, 2016; Jimenez-Pacheco et al., 2016; Volonte et al., 2016; Cieslak and Wojtczak, 2018; Cieslak et al., 2019). Yet, preclinical data in mouse models of ALS leave some uncertainty about it, as previously described. Moreover, P2X7R antagonists have not succeeded in any CT for CNS disorders so far (ClinicalTrials.gov - NIH U.S. National Library of Medicine, 2020). Despite the notable number of P2X7R blockers already existing as pharmacological tools, not all meet the pharmacokinetic (PK) characteristics that are necessary to become a potential drug for the treatment of neurodegenerative diseases, including ALS. This is the case of BBG that has been amply used as a P2X7R blocker only because it is cheap and readily available. However, this compound has a poor selectivity towards P2X7Rs, blocking also P2X4, P2X5, and rat P2Y1 and P2Y2, while potentiating the human P2Y1 at 1–3 μM concentrations (Jacobson et al., 2002, 2006; Jacobson, 2010). Research has led nowadays to very potent antagonists, but unfortunately, this is not enough. Between the main problems of these compounds, there is a lack of favorable PK properties, such as low blood–brain barrier (BBB) permeability and scarce stability in blood and brain tissue, which determine a drop in drug half-life and CNS residence time. Additionally, pronounced differences have been found in potencies and affinities among P2X7R in mouse, rat, and human. Such is the case of AZ11645373 (Figure 1) that blocks the human P2X7R at nanomolar concentrations and the mouse P2X7R at micromolar concentrations and has no effect on the rat orthologous (Michel et al., 2009). Another example is compound GW791343, which is a potent allosteric inhibitor of the human receptor, but also a positive allosteric modulator of rat P2X7R (Jacobson, 2010). This complicates the scenario for the development of a drug for clinical use and diminishes the possibility for a new compound of being selected for preclinical and clinical studies.

During around the last 10–15 years, the interest in finding new P2X7R clinical candidates has shifted from no-BBB-permeable antagonists for the treatment of peripheral inflammatory diseases (e.g., **AZD-9056** from AstraZeneca, tried in CTs—Figure 1) to BBB-permeable blockers, mainly due to the increasing awareness of P2X7R role in CNS disorders (Bhattacharya and Biber, 2016; Bhattacharya, 2018).

However, just increasing lipophilicity is not always highly recommended for the development of BBB-permeable drugs. In fact, this can penalize other important PK properties like solubility, enzyme metabolism, blood protein binding, and half-life. After the development of the high lipophilic *o-*chlorobenzamide P2X7R antagonists by Pfizer, their attempts were focused on the increase of molecular polarity, giving a good clinical candidate that, however, was no longer CNS penetrant (**CE-244,535**—Figure 1; Duplantier et al., 2011).

**Figure 2:**
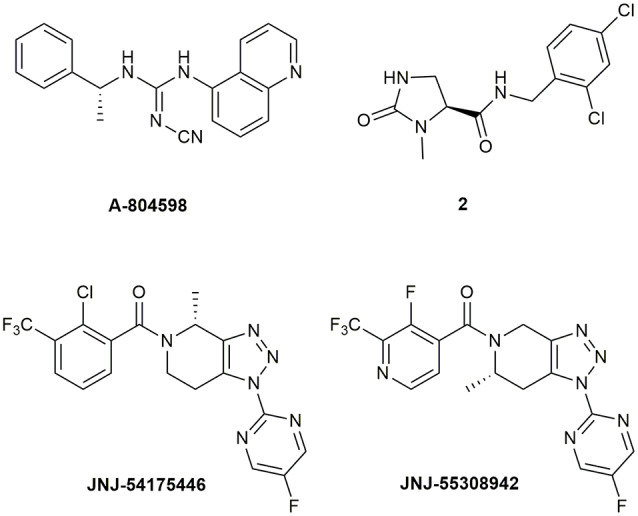
P2X7R antagonists with optimal pharmacokinetic properties for the treatment of CNS disorders.

Abbott Laboratories developed several potent P2X7R antagonists, mainly tetrazoles and cyanoguanidines. Thus, tetrazole derivative **A-438079** (Figure 1) was very appealing for its small molecular weight, good potency, and efficacy in reverting allodynia in the Chung model of neuropathic pain. Nevertheless, its half-life of 1 h and low bioavailability after intraperitoneal (ip) administration was discouraging (Guile et al., 2009). Cyanoguanidine **A-740003** (Figure 1) was found to have a better half-life after iv administration (*t_½_* = 4 h) but not a good BBB permeability, as confirmed by its radiotracer [^11^C]A-740003 (Janssen et al., 2014). Cyanoguanidine **A-804598** (Figure 2) has the best balance of pharmacodynamics and PK properties. In fact, it is potent (IC_50_ of around 10 nM for mouse, rat, and human species, vs. the potency of A-740003 ranging from 44 to 150 nM in the different orthologs), selective for the P2X7R, and brain-permeable, persisting in brain tissues at least for 1 h after administration, as shown in *ex vivo* studies of receptor occupancy (Able et al., 2011). More recently, Abbott has developed new potent P2X7R antagonists by adding the adamantine core to cyanoguanidines (compound **1**—Figure 1), but without great PK improvements (e.g., *t*_½_ = 0.22 h; O’Brien-Brown et al., 2017).

GlaxoSmithKline developed a series of compounds with excellent physicochemical properties, such as the BBB-permeable **GSK-1482160** in rats (Figure 1; Abdi et al., 2010). Unfortunately, deeper studies on its metabolism pathway explained why some parameters, like its half-life, worsened in dog and monkey models, blocking its clinical development. Anyway, new chemical modifications succeeded in overcoming this problem. Among the compounds of this new series, the oxoimidazolidine **2** (Figure 2) stands out (Abberley et al., 2010).

It is a fact that Janssen Pharmaceuticals has probably developed the most interesting P2X7R antagonists as clinical candidates for the treatment of CNS diseases. One of the most used ligands in basic and preclinical research is **JNJ-47965567** (Figure 1). Unfortunately, its poor oral bioavailability, as well as the lack of deeper details on its residence time in brain, stopped its clinical progress (Bhattacharya et al., 2013). However, new series of dihydro- and tetrahydro-triazolopyridines were discovered and extensively optimized. Among these, **JNJ-54175446** and **JNJ-55308942** (Figure 2) have optimal PK properties like solubility, metabolism profile, bioavailability, and half-life, as well as good BBB permeability and tolerability (Letavic et al., 2017; Chrovian et al., 2018). Proof of this is the fact that they were chosen as clinical candidates and that **JNJ-55308942** has completed three phase I CTs, while **JNJ-54175446** is currently in a phase II CT for major depression (ClinicalTrials.gov - NIH U.S. National Library of Medicine, 2020; Recourt et al., 2020).

## Proof of Concept of P2X7Rs as a Druggable Target in ALS

The proof of concept that a P2X7R antagonist could have promising therapeutic potential in ALS has been tested in the SOD1^G93A^ mouse model of ALS (Table 1). In a first study, BBG was administered intraperitoneally at a dose of 45.5 mg/kg every 48 h, starting at postnatal day 90 (P90), a late pre-onset disease stage. The treatment improved deficits in motor performance to a greater extent in males than in females, but no effect on survival was observed (Cervetto et al., 2013).

**Table 1:** Proof of concept of P2X7R antagonism on clinical outcomes in SOD1^G93A^ mice.

Reference	Treatment	Treatment starting point	Main outcomes
Cervetto et al. (2013)	BBG, 45.5 mg/kg, every 48 h, ip	P90 (pre-onset) to humane end point
			Improvement in motor performance in both genders, although greater effect in males. Delayed weight loss in males. No difference in survival was observed.
Apolloni et al. (2014)	BBG, 50 mg/kg, three times/week, ip	P100 (late pre-onset) and P135 (onset) to humane end point	Improvement in motor performance in mice treated from onset. Disease onset and survival not affected.
	BBG, 250 mg/kg, three times/week, ip	P40 (asymptomatic), P70 (pre-onset), P100 (late pre-onset) to humane end point	Improved behavioral scores and motor performance in mice treated from late pre-onset. No differences in survival. Decrease in microgliosis, inflammatory markers and motor neuron loss in late pre-onset treated animals.
Bartlett et al. (2017)	BBG, 45.5 mg/kg, three times/week, ip	P60 (pre-onset) to humane end point	Reduced body weight loss and prolonged survival in females. No effect on clinical score or motor performance
Fabbrizio et al. (2017)	A-804598, 30 mg/kg, five times/week, ip	P100 (pre-onset) to humane end point	No effect on motor performance, behavioral scores or survival observed
Ly et al. (2020)	JNJ-47965567, 30 mg/kg, three times/week, ip	P100 (onset) to humane end point	No effect on motor performance, ALS score or survival observed. No altered gene expression in spinal cord. No altered proportions of lymphoid leukocytes. No effect on serum cytokines.

Taking into account the dual role of P2X7Rs (protection at early stages and promotion of cell death at advanced disease stages), an experiment was designed changing the dose and defining different start points of BBG administration (250 mg/kg, 3 times/week). Starting at late pre-onset stage (P100), higher MN survival and reduced microgliosis were observed in the spinal cord of SOD^G93A^ mice. Moreover, disease onset was delayed and motor performance was improved in both males and females, although survival was not affected. This was not the case when BBG was administered at pre-symptomatic stages, supporting the hypothesis of the dual role of P2X7R in ALS (Apolloni et al., 2014).

In a third experiment, BBG administration at pre-onset stage or P62–P64 (45.5 mg/kg, three times/week) reduced body weight loss in females but not in males, a sign of delayed muscle loss. Survival was also augmented in females but not in males, and motor performance was unaffected in either sex (Bartlett et al., 2017).

Finally, two experiments have used more potent and specific P2X7R antagonists. When the antagonist A-804598 was administered to female mice five times/week at 30 mg/kg, no effects on motor performance, disease onset, or survival were observed (Fabbrizio et al., 2017). Same negative results were obtained with the administration of the antagonist JNJ-47965567 from P100, three times/week at 30 mg/kg (Ly et al., 2020).

These erratic, yet mild positive outcomes suggest that the experimental conditions of future experiments should be considered as follows: (i) to adjust drug administration starting point; (ii) to define better the dosing and frequency of administration, according to (iii) the PK drug properties, including the BBB permeability and CNS residence time, longer half-life and solubility; and (iv) to include both females and males and, if possible, different ALS mouse models.

## From Bench to Clinical Trials

Recent reviews (Petrov et al., 2017; Andrews et al., 2019; Chipika et al., 2019) analyze the outcomes of over 50 CTs performed in ALS patients, since riluzole was available. Various druggable potential targets have been addressed: (1) antiglutamatergic compounds (riluzole, memantine, talampanel, ceftriaxone); (2) antioxidant agents (edaravone, coenzyme Q, creatine); (3) anti-inflammatory drugs (valproic acid, NP001, glatiramer acetate, minocycline, pioglitazone, erythropoietin, celecoxib); (4) neurotrophic factors (IGF-1, CNTF, BDNF); (5) lithium; (6) inhibitors of kinase (masitinib, fasudil); and (7) neuroprotective compounds (xaliproden, dexpramipexole, olesoxime, omigapil).

Despite riluzole and edaravone being approved by the FDA after having been tested in CTs, they gave poor clinical outcomes. In the case of riluzole, two out of three late-stage CTs reported negative outcomes. Similarly, in the case of edaravone, two out of three phase III CTs also reported negative results. The rest of the compounds and drugs tested provided negative results in CTs.

Masitinib is emerging as a singular agent to mitigate microgliosis and neuroinflammation. The compound also prolongs survival when administered to SOD1^G93A^ ALS mice at the onset of paralysis (Trias et al., 2016). An add-on riluzole therapy phase III CT reported significant outcomes in ALS patients treated with masitinib [Masitinib in Combination With Riluzole for the Treatment of Patients Suffering From Amyotrophic Lateral Sclerosis (ALS), 2018].

## Conclusions and Perspectives

Much is known about clinical features and pathogenic pathways of ALS. Cumulating knowledge, derived from *in vitro* and *in vivo* disease models, as well as from patients and postmortem tissues, outlines the sequence of the pathophysiological events involved in ALS. These could be hypothetically ordered as follows: (1) cytosolic and mitochondrial protein aggregation, affecting mitochondrial ultrastructure and function; (2) distorted mitochondrial calcium handling and circulation; (3) deficits in ATP generation and excess of ROS production; (4) reactive microglia and production of inflammatory mediators; and (5) enhanced MN vulnerability and death.

Regarding disease pathogenesis, a crucial open question remains: are the pathogenic features common for both sporadic and familial ALS? And also, why do only MNs die, if mutated proteins are expressed in all cell types? Given the complexity of ALS pathogenesis, why have CTs been performed using single-target compounds in the last 20 years? In light of the chronic inflammatory background, should we target with neuroprotective agents only the MNs, or the activated glia, or both? Much further research is required before we can answer these questions.

## Author Contributions

CR-R and FC contributed equally to this manuscript. They both searched for scientific literature concerning ALS pathogenesis and P2X7R involvement. CR-R reviewed the most considerable ALS mouse models used in research, while FC made a summary of the molecular and pharmacokinetic profiles of the main P2X7 receptor blockers. AG re-elaborated and drafted the final form of the manuscript, reviewing also the subsequent versions.

## Conflict of Interest

The authors declare that the research was conducted in the absence of any commercial or financial relationships that could be construed as a potential conflict of interest.

## Abbreviations

[Ca^2+^]_c_: Ca^2+^ concentration in the cytosol
ALS: amyotrophic lateral sclerosis
AMPA: α-amino-3-hydroxy-5-methyl-4-isoxazolepropionic acid
BBB: blood–brain barrier
BBG: Brilliant Blue G
BDNF: brain-derived neurotrophic factor
BzATP: 3’-O-(4-benzoyl)benzoyl-ATP
C9orf72: Chromosome 9 open reading frame 72
CBPs: calcium-binding proteins
CNS: central nervous system
COX-2: cyclooxygenase-2
CSF: cerebrospinal fluid
CT: clinical trial
EAAT2: excitatory amino acid transporter 2
fALS: familial ALS
FDA: U.S. Food and Drug Administration
FUS/TLS: Fused in Sarcoma/Translocated in Sarcoma
GDNF: glia cell-derived neurotrophic factor
H_2_O_2_: hydrogen peroxide
HIV: human immunodeficiency virus
ip: intraperitoneal
iv: intravenous
MCU: mitochondrial Ca^2+^ uniporter
mNCX: mitochondrial Na^+^/Ca^2+^ exchanger
MN: motor neuron
NMDA: N-methyl-D-aspartate
NOX: NADPH oxidase
O_2_^−^: superoxide radical
OXPHOS: mitochondrial oxidative phosphorylation system
P2X7R: purinergic P2X7 receptor
P90: postnatal day 90
PET: positron emission tomography
PK: pharmacokinetics
R: receptor
RAN: repeat-association non-ATG
ROS: reactive oxygen species
sALS: sporadic ALS
SOD1: Cu/Zn superoxide dismutase
TAR: transactive response
TDP-43: TAR-DNA binding protein-43
VACCs: voltage-activated calcium channels
VEGF: vascular endothelial growth factor
WT: wild type.
